# Is a prostate cancer screening anxiety measure invariant across two different samples of age-appropriate men?

**DOI:** 10.1186/1472-6947-12-52

**Published:** 2012-06-08

**Authors:** Suzanne K Linder, Paul R Swank, Sally W Vernon, Robert O Morgan, Patricia D Mullen, Robert J Volk

**Affiliations:** 1 Department of General Internal Medicine, The University of Texas MD Anderson Cancer Center, Houston, TX, USA; 2 School of Public Health, The University of Texas Health Science Center, Houston, TX, USA; 3 School of Medicine, The University of Texas Health Science Center, Houston, TX, USA

## Abstract

**Background:**

In order to explore the influence of anxiety on decision–making processes, valid anxiety measures are needed. We evaluated a prostate cancer screening (PCS) anxiety scale that measures anxiety related to the prostate–specific antigen (PSA) test, the digital rectal examination (DRE), and the decision to undergo PCS (PCS-D) using two samples in different settings.

**Methods:**

We assessed four psychometric properties of the scale using baseline data from a randomized, controlled decision aid trial (n = 301, private clinic; n = 149, public).

**Results:**

The 3-factor measure had adequate internal consistency reliability, construct validity, and discriminant validity. Confirmatory factor analyses indicated that the 3–factor model did not have adequate fit. When subscales were considered separately, only the 6–item PCS-D anxiety measure had adequate fit and was invariant across clinics.

**Conclusions:**

Our results support the use of a 6–item PCS-D anxiety measure with age-appropriate men in public and private settings. The development of unique anxiety items relating to the PSA test and DRE is still needed.

## Background

Patient decision aids are recommended for healthcare decisions for which there is no single best evidence–based option and personal preferences dictate the best option [1,2]. Decision aids provide balanced information, help clarify patient values, and facilitate the weighing the risks and benefits against a preferred set of personal criteria [1,3]. Whether to receive prostate cancer screening (PCS) is such a decision in that the best choice relies on the patient’s preferences for the various risks and benefits [3,4]. Indeed, national organizations that provide PCS guidelines recommend physicians to discuss the risks and benefits of screening and treatment and to take patients’ personal preferences into account [5-9]. Decision aids are strongly recommended to facilitate PCS decision-making because several treatments for prostate cancer may cause unnecessary physical and psychological harm [10-14] and because randomized controlled trials have shown that the prostate–specific antigen (PSA) test and digital rectal examination (DRE) provide little or no benefit in the early detection of prostate cancer by reducing the prevalence of deaths due to the disease [11,13].

The influence of affect on cognitive decision–making processes has been largely ignored in decision aid studies. Because the affect construct of anxiety is known to influence effective decision–making, especially in decisions that involve uncertainty [15-17], some researchers have recommended exploring the moderating role of anxiety on cognitive decision–making processes [18]. Anxiety may moderate the effect of decision aids as well. Anxiety is one of the most widely used affect constructs in decision aid studies but has usually been conceptualized as an outcome rather than a moderator of a decision [1,19-23].

Researchers have called for decision–specific measures to be used in decision aid studies [24,25]. Such measures are necessary because healthcare decisions vary in social context; patients may vary in the degree to which they place personal importance on the specific decision, the different types of options, and the uncertainty among the options, risks, and benefits [26]. Furthermore, healthcare decisions differ in their potential consequences, in decision needs that must be addressed for informed decision making to occur, and in timing [27]. In decision aid studies, anxiety is usually conceptualized as being a general, stable trait instead of anxiety specifically related to aspects of the healthcare decision. For these reasons, decision–specific anxiety measures are needed in order to evaluate the role of this affect on cognitive decision–making processes.

In this study, we tested a PCS anxiety scale to determine the measure’s psychometric properties across to different samples of age-appropriate men. In order to generalize and compare the effect of PCS anxiety on cognitive decision-making processes across different populations and settings, the measure’s structure must be invariant. Without measurement invariance, interpretations across samples and settings are not valid. This secondary analysis used baseline data from a randomized, controlled PCS decision aid trial that had evaluated two decision aids, one at a private clinic and the other at a public clinic [28]. We assessed the reliability, construct validity, and discriminant validity for the PCS anxiety instrument and determined its latent structure and invariance across settings.

## Methods

### Development of the PCS anxiety instrument

Two authors (RJV, SKL) originally constructed a 21–item PCS anxiety measure using wording similar to that in the Spielberger State–Trait Anxiety Inventory [29]. Three subscales were created using similar wording for the subscale items. Two subscales related to anxiety about the screening tests (PSA and DRE subscales), and the third represented anxiety related to the decision about undergoing screening for prostate cancer (PCS-D subscale). For initial determination of content validity and appropriate word choice, we conducted individual cognitive interviews with five men each from the public clinic and the private clinic. As a result of these interviews, one item from each proposed subscale was discarded and the statement format was changed to a question format.

The final PCS anxiety measure consisted of 18 questions, with 6 items each in three subscales (Table 1). Each item had ordered categorical response options of “yes” (4 points), “no” (0 points), and “unsure” (2 points). One negatively framed item (“relaxed”) in each subscale was reverse coded. For the overall PCS anxiety score, the 18 items were summed, divided by the number of items, and multiplied by 25. The possible range for the overall value was 0 (no anxiety) through 100 (extremely high anxiety). The PSA, DRE, and PCS-D anxiety subscale scores were created in a similar fashion.

**Table 1:** Questions from the prostate cancer screening anxiety measure

**PSA anxiety**			
**For having a blood test for cancer…**	**Yes**	**No**	**Not Sure**
Do you feel tense about the test?	[ ]	[ ]	[ ]
Do you feel upset about the test?	[ ]	[ ]	[ ]
Do you feel nervous about the test?	[ ]	[ ]	[ ]
Do you feel confused about the test?	[ ]	[ ]	[ ]
Do you feel worried about the test?	[ ]	[ ]	[ ]
Do you feel relaxed about the test?	[ ]	[ ]	[ ]
**DRE anxiety**			
**For having a test for cancer where the doctor or nurse inserts a finger into your rectum to perform an exam…**			
Do you feel tense about the exam?	[ ]	[ ]	[ ]
Do you feel upset about the exam?	[ ]	[ ]	[ ]
Do you feel nervous about the exam?	[ ]	[ ]	[ ]
Do you feel confused about the exam?	[ ]	[ ]	[ ]
Do you feel worried about the exam?	[ ]	[ ]	[ ]
Do you feel relaxed about the exam?	[ ]	[ ]	[ ]
**PCS-D anxiety**			
**For the decision to be tested for prostate cancer…**			
Do you feel tense about the decision?	[ ]	[ ]	[ ]
Do you feel upset about the decision?	[ ]	[ ]	[ ]
Do you feel nervous about the decision?	[ ]	[ ]	[ ]
Do you feel confused about the decision?	[ ]	[ ]	[ ]
Do you feel worried about the decision?	[ ]	[ ]	[ ]
Do you feel relaxed about the decision?	[ ]	[ ]	[ ]

*Note.* PCS = prostate cancer screening, PSA = prostate–specific antigen test, DRE = digital rectal examination.

### Participants and procedures

We used de–identified baseline data from a randomized controlled trial that had compared the effects of a computerized PCS decision support tool and an active control tool (audio information booklet) [28]. Between January 2004 and February 2006, a total of 450 men had been recruited for the study. These study participants had been scheduled for non–acute primary care appointments, had no history of prostate cancer, were 50–70 years of age (40–70 years of age if African–American), and had not had a PSA test within the previous 6–12 months. Recruitment occurred at two sites in Houston, Texas: a general medicine clinic at a publicly funded hospital (n = 149) and a private, university–affiliated primary care clinic (n = 301). The following self–reported variables from the trial were used in our study: 1) socio–demographic characteristics (i.e., age, ethnicity/race, education, health status, insurance status, and family history of prostate cancer), 2) screening intention, 3) clinic site, and 4) PCS anxiety level. Screening intention had been indicated by the response to the question, “Given what you know about prostate cancer and PSA testing, do you plan to have a PSA test?” with response options “yes,” “no,” “not sure.” The study sample characteristics are described elsewhere [28]. Our study was approved by The University of Texas School of Public Health Research Service Center and exempted from review by the Committee for the Protection of Human Subjects. The original trial had been approved by the Baylor College of Medicine Institutional Review Board and the Harris County Hospital District.

### Data analysis

SPSS version 16.0 was used for descriptive analyses, to estimate internal consistency reliability, and to estimate construct and discriminant validity. The chi-square (*χ*^2^) test was used to assess differences in demographic characteristics across clinic sites. Mplus version 5.1 was also used for internal consistency reliability and for all factor analyses.

#### Internal consistency reliability

Internal consistency reliability was evaluated using intraclass correlation coefficients from factor analysis to account for multiple weights. We expected coefficients ≥ .40. Internal consistency reliability for the overall scale and the three subscales for the total sample and by clinic site is reported using Cronbach’s α, which we expected to be ≥ .70, the recommended minimum value for acceptable internal consistency reliability for research or group comparisons [30].

#### Construct validity

To assess the construct validity of the PCS anxiety instrument, we examined patterns of correlations among the subscales by using a one–tailed Pearson correlation with p ≤ .05 indicating statistical significance. We hypothesized that the PCS-D anxiety subscale would be positively and substantially correlated with the two testing procedure anxiety subscales (DRE and PSA subscales). In other words, if anxiety about the PSA test or DRE were high, then anxiety about the PCS decision would also be high. We expected a weaker correlation between the PSA and DRE anxiety subscales because the two tests are administered differently (i.e., blood drawn for testing versus rectal examination). We also expected men to be more anxious about the DRE than the PSA test, as would be indicated by higher mean scores for the DRE anxiety subscale.

#### Discriminant validity

To determine whether the PCS anxiety measure can differentiate between groups of men based on screening intention, the total mean scores of this measure were compared using two-way analysis of variance (ANOVA) with p ≤ .05 considered statistically significant. The independent variables were clinic site and two screening intention contrasts. The contrasts were created to compare men who had made a decision about undergoing screening (yes or no) and to compare men who had made a decision with those who had not (decided versus unsure). To determine whether the three PCS anxiety subscales could differentiate groups of men based on screening intention, a repeated-measures ANOVA was performed with the mean scores of each subscale entered as dependent variables. We hypothesized that men who were undecided would have higher PCS anxiety total and subscale scores than men who had made a decision regarding screening.

#### Factor validity

**Figure 1:**
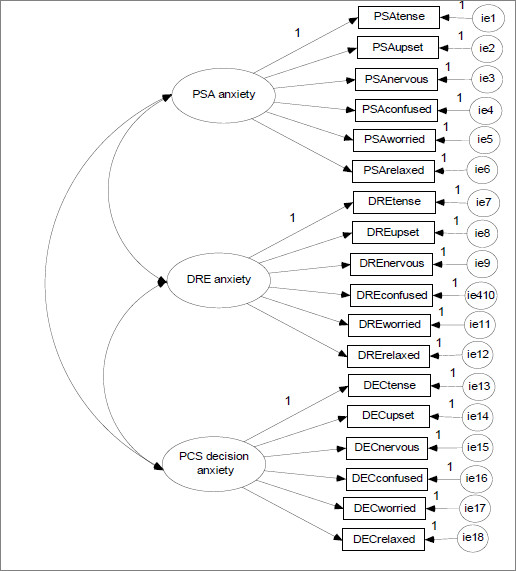
**Prostate cancer screening anxiety 3–factor model.***Note.* PCS = prostate cancer screening, PSA = prostate–specific antigen test, DRE = digital rectal examination.

Single–group confirmatory factor analyses (CFAs) were conducted to assess model fit for the proposed 3–factor model (Figure 1). We used a mean– and variance–adjusted weighted least squares estimator (WLSMV) because the data were ordered categorically and the responses to the anxiety items were non–normally distributed [31]. This robust estimation of standard errors and robust *χ*2 tests of model fit take into account non–normality of outcomes and non–independence of observations due to cluster sampling [31].

Model fit was assessed by examining the *χ*2 test of model fit, the root mean square error of approximation (RMSEA), the comparative fit index (CFI), the Tucker–Lewis index (TLI), and the weighted root mean square residual (WRMR). A combination of a statistically non–significant *χ*2 value (p ≥ .05), RMSEA < .05, CFI and TLI > .95, and WRMR < .90 indicate adequate model fit [32,33].

#### Factor invariance

After finding an acceptable fit based on the single–group CFA, a two–group CFA was performed to determine measurement invariance across clinic sites. Two models were created based on models for measurement invariance of categorical outcomes using WLSM with delta parameterization: 1) less restrictive: threshold and loading factors free across groups, scale factors fixed at one in all groups, and factor means fixed at zero in all groups, and 2) more restrictive: thresholds and factor loadings constrained to be equal across groups, scale factors fixed at one in one group and free in others, and factor means fixed at zero in one group and free in others [31]. We calculated the *χ*2 difference between the two models based on difference testing for WLSM using DIFFTEST in Mplus [31]. A non–significant *χ*2 difference value (p > .05) indicated measurement invariance.

## Results

### Internal consistency reliability

All internal consistency reliability coefficients were acceptable as indicated by Cronbach’s α ≥ .70 (total PCS anxiety measure, α = .910; PSA anxiety subscale α = .830; DRE anxiety subscale α = .819; PCS-D anxiety subscale α = .834). All internal consistency reliability coefficients were higher for men at the public clinic than at the private clinic but not appreciably so (total PCS anxiety measure α = .927 vs. .895; PSA anxiety subscale α = .837 vs. .826; DRE anxiety subscale α = .857 vs. .794; PCS-D anxiety subscale α = .869 vs. .788). Intraclass correlation coefficients are not presented because of the lack of fit found for the 3-factor model.

### Construct validity

All the three anxiety subscales were positively correlated and were statistically significant (range, r = .513 – .790, p < .001 for all comparisons) (Table 2). Following the same pattern as the internal consistency reliability coefficients, Pearson correlation coefficients for the anxiety subscales were higher for men at the public clinic than those at the private clinic. As we hypothesized, the two testing procedure subscales were positively correlated with the PCS-D anxiety subscale and were more strongly correlated with it than with each other. This finding held for the total sample as well as for both clinics.

**Table 2:** Means and correlations coefficients for the prostate cancer screening anxiety

	**Means (SD)**			Correlation Coefficients*					
	**Total**	**Public Clinic**	**Private Clinic**	**Total**		**Public Clinic**		**Private Clinic**	
				**DRE**	**PCS-D**	**DRE**	**PCS-D**	**DRE**	**PCS-D**
PCS anxiety total scale	20.15 (23.43)	23.95 (26.99)	18.25 (21.35)	---	---	---	---	---	---
PSA anxiety subscale	17.62 (26.41)	18.65 (27.61)	17.11 (25.83)	.517	.682	.525	.790	.513	.618
DRE anxiety subscale	26.88 (29.85)	30.31 (33.21)	25.28 (27.97)	---	.642	---	.680	---	.611
PCS-D anxiety subscale	15.95 (25.27)	22.13 (30.75)	12.84 (21.38)	---	---	---	---	---	---

^*^All Pearson correlation coefficients were significant at p ≤ 0.01 (1–tailed).

*Note.* SD =standard deviation, PCS = prostate cancer screening, PSA = prostate–specific antigen test, DRE = digital rectal examination, PCS-D = prostate cancer screening decision.

The mean anxiety scores for the total scale and three subscales were relatively low, ranging from 12.84 to 30.31 out of 100 (Table 2). As we expected, men at both clinics were more anxious about the DRE than the PSA test.

### Discriminant validity

ANOVA results partially supported our hypothesis that men who were undecided would have higher PCS anxiety total and subscale scores than men who had made a decision regarding screening. The total PCS anxiety scores can discriminate groups of men based on screening intention (decided versus unsure) at the public clinic (Table 3). At the public clinic, men who were unsure about their screening intention had significantly higher total PCS anxiety scores (mean = 35.19) than men who had made a decision about their screening intention (mean = 20.25). At the private clinic, however, the difference in total PCS anxiety score was not significant (decided, mean = 17.94; unsure, mean = 20.25).

**Table 3:** Summary of analysis of variance for clinic site and screening intention contrasts

**Source**	**Sum of Squares**	**df**	**Mean Square**	**F**	**P–Value**
Clinic site	443.012	1	443.012	.834	.361
si1	64.799	1	64.799	.122	.727
si2	3,089.853	1	3,089.853	5.820	**.016**
site * si1	457.553	1	457.553	.862	.354
site * si2	2,687.349	1	2,687.349	5.062	**.025**
Error	227,220.053	428	530.888		
Total	410,817.901	434			
Corrected Total	236,416.980	433			

*Note.* df = degrees of freedom; si1 = screening intention (yes versus no); si2 = screening intention (decided versus undecided); bold font indicates statistical significance (p < .05).

### Factor validity

CFAs were conducted separately for each clinic site. The proposed 3–factor model did not have adequate fit for either site (Table 4). For the private clinic, inadequate fit was indicated by the *χ*^2^, CFI, RMSEA, and WRMR values. For the public clinic, inadequate fit was indicated by the *χ*^2^ and RMSEA values.

**Table 4:** Confirmatory factor analyses of the 3–factor prostate cancer screening anxiety model by clinic site

**Model**	**Chi–square (df)**	**P–value**	**CFI**	**TLI**	**RMSEA**	**WRMR**
Private Clinic						
3-Content	178.932 (38)	<.001	.921	**.960**	.112	1.369
3-Content + 6-Method	50.999 (36)	**.050**	**.992**	**.996**	**.038**	**.543**
Public Clinic						
3-Content	71.563 (31)	<.001	**.967**	**.985**	.095	**.884**
3-Content + 6-Method	26.565 (29)	**.595**	**1.000**	**1.001**	**<.001**	**.420**

*Note.* Content = hypothesized subscales; Method = similarly worded items; CFI = comparative fit index; TLI = Tucker–Lewis index; RMSEA = root mean square error of approximation; WRMR = weighted root mean square residual; bold font indicates adequate fit.

Exploratory factor analyses were then conducted to examine factor loadings, which indicated some evidence for content factors (i.e., PSA, DRE, and PCS-D) and for method factors. The method factors consisted primarily of similarly worded items across the subscales. Based on the factor loadings, a CFA model with 3 content and 6 method factors was completed. Although all indices indicated adequate model fit for both clinics, over–fitting the model became an issue (9 factors with 18 observed variables). Additionally, two warnings were given: the residual covariance matrix (theta) and the latent variable covariance matrix (psi) were not positive definite. These warnings indicated a negative variance/residual variance for an observed variable and a latent variable, respectively [31]. When we fixed these variances to 0, the model fit was worse as indicated by the fit indices and additional warnings resulted for other items and latent variables. Therefore, we concluded that the 3–content and 6–method model was not appropriate.

Three 1–factor CFAs were then completed to separately analyze anxiety related to the PSA test, DRE, and PCS-D. For both clinic sites the 1–factor models for PSA anxiety and PCS-D anxiety fit adequately according to all fit indices (Table 5). In contrast, the 1–factor model for DRE anxiety had adequate model fit at the public clinic, according to all fit indices, but had poor fit at the private clinic, according to the *χ*^2^ and RMSEA results.

**Table 5:** Confirmatory factor analysis for the 1–factor anxiety models

**Model**	**Chi–square (df)**	**P–value**	**CFI**	**TLI**	**RMSEA**	**WRMR**
1–Factor PSA anxiety						
Private Clinic	6.810 (7)	**.4489**	**1.00**	**1.00**	**<.001**	**.379**
Public Clinic	7.002 (6)	**.3207**	**.999**	**.999**	**.034**	**.382**
1–Factor DRE anxiety						
Private Clinic	29.662 (7)	.0001	**.983**	**.983**	.105	**.863**
Public Clinic	4.334 (7)	**.7406**	**1.000**	**1.004**	**<.001**	**.305**
1–Factor PCS-D anxiety						
Private Clinic	7.154 (6)	**.3068**	**.999**	**.999**	**.026**	**.449**
Public Clinic	8.776 (7)	**.2691**	**.998**	**.999**	**.042**	**.373**

*Note.* df = degrees of freedom; PSA = prostate–specific antigen test, DRE=digital rectal examination; PCS-D = prostate cancer screening decision; CFI = comparative fit index; TLI = Tucker–Lewis index; RMSEA = root mean square error of approximation; WRMR = weighted root mean square residual; bold font indicates adequate fit.

### Factor invariance

Finally, to evaluate invariance between the clinics, we conducted two–group CFAs for a 1–factor PSA anxiety measure and a 1–factor PCS-D anxiety measure. The *χ*^2^ difference test indicated non–invariance for the first measure (p < .0001), and invariance for the second measure (p = .0889) (Table 6). Comparison of factor loadings across clinics showed a similar pattern for all six items. Except for one item (relaxed, reversed coded), the factor loadings were larger for the private clinic than for the public clinic. Examination of the categorical item frequencies revealed some differences in the response distributions, with men at the public clinic more likely to use respond “no” (anxious) than men at the private clinic were, but that most men at both clinics reported “yes” (not anxious) for all items.

**Table 6:** Measurement invariance for the two 1–factor anxiety models

	**Chi–square (df)**	**P–value**	**CFI**	**TLI**	**RMSEA**	**WRMR**
1–Factor PSA anxiety						
1. less restrictive	13.052 (13)	**.4438**	**1.00**	**1.00**	**.004**	**.560**
2. more restrictive	34.804 (12)	.0005	**.989**	**.988**	.092	1.225
1. vs 2.	23.312 (4)	.0001				
1–Factor PCS-D anxiety						
1. less restrictive	14.206 (12)	**.2878**	**.999**	**.999**	**.029**	**.577**
2. more restrictive	23.553 (16)	**.0997**	**.996**	**.997**	**.046**	**.833**
1. vs 2.	9.554 (5)	**.0889**				

*Note.* df = degrees of freedom; PSA = prostate–specific antigen test, DRE=digital rectal examination; PCS-D = prostate cancer screening decision; CFI = comparative fit index; TLI = Tucker–Lewis index; RMSEA = root mean square error of approximation; WRMR = weighted root mean square residual; bold font indicates adequate fit.

## Discussion

To our knowledge, this study is the first attempt to assess the factor validity and invariance of an anxiety measure used in decision aid trials. We used cognitive interviewing to refine the anxiety items, and we used factor analyses to evaluate factor validity and invariance. We also demonstrated the internal consistency reliability, the construct validity, and the discriminate validity by using various psychometric testing methods. Our findings from CFA support the use of a 1–factor, 6-item general anxiety measure for the decision to undergo PCS as a potential moderator in PCS decision aid studies that is appropriate to be used in private and public settings. The validity of the PSA and DRE subscales is uncertain.

Our results also provide partial support for the use of a 1–factor PSA anxiety measure. Although the measure was non-invariant across clinics, this result may not be clinically meaningful in this instance because the factor loading patterns were similar in direction and only slightly different in magnitude. Moreover, the small sample size for the public clinic (n = 149) may have affected the accuracy and precision of our measurement invariance testing [34]. In addition, the factor loading estimates may be biased due to truncated distributions for the private clinic sample: categorical item frequencies indicated that men at the private clinic were less likely to report “no” (highest anxiety category) than men at the public clinic. This difference could be due to their history of PSA testing: men at the private clinic were more likely to report prior PSA testing than men at the public clinic were (70.4% versus 30.9%).

Our study results support the use of structural equation modeling techniques when psychometric properties of measures are being evaluated. Although our initial tests for internal consistency reliability and discriminant and construct validity indicated some evidence for the 3–factor model, factor analyses indicated a high degree of inter–correlation between items across factors. There was no evidence of discriminant validity to indicate that the items were three separate subscales. Many items loaded on factors other than those proposed, and there was a high degree of method variance. Some items seemed to be mostly related to method variance, some were mostly related to content, and some seemed to share variance with factors that were not conceptually identifiable.

The reported low levels of anxiety at both clinics may also have contributed to the lack of fit for the 3-factor PCS anxiety measure. Low variability can cause difficulty in distinguishing between factors. The low anxiety levels might be due to the prevalence of recent PSA testing. The low levels could also be due to the nature of the decision: men may not be anxious in general about PCS because messages about screening are that it is almost always beneficial [35].

On the basis of our results, we can suggest recommendations for future development of decision-specific anxiety measures. First, our findings that two of the three PCS subscales were non-invariant across the two samples that varied by clinic setting exemplify the importance to evaluate measurement invariance before comparing scale scores across samples. When measurement non-invariance is found, there may be indication that the samples differ in their underlying meaning of the anxiety construct. Therefore, comparisons of the scores across the samples may not be meaningful. Second, developing decision-specific measures may need more formative work (i.e., cognitive testing or focus groups) to explore the anxiety specifically related to the healthcare decision. For PCS, it may be necessary to understand what makes men anxious about the screening decision and the testing procedures: some men may be worried about the type of tests (blood drawn with a needle or rectal examination), while others may be anxious about the test results or accuracy. Third, we suggest avoiding using the same wording across subscales to minimize method variance. Finally, we recommend not to include negatively framed items in factor analyses. During cognitive interviewing, we discarded one negatively framed item (“calm”) that men did not interpret as the opposite of being anxious However, we included one negatively framed item (“relaxed”) in each subscale (3 items total) in our PCS anxiety measure. Two of these three negatively framed items loaded as a separate factor and not on the respective subscale factor.

Our findings have several limitations. First, we did not measure situational (state) anxiety or underlying (trait) anxiety. It would be helpful to know how PCS anxiety, a context–specific anxiety, is different than one’s general anxiety. Although the comparison groups were based on clinic site, one serving primarily privately insured patients and the other primarily publicly insured patients, there were statistically significant differences between the men at each clinic (i.e., race/ethnicity, recent history of PSA testing, education, and insurance status). Our results may not be generalizable to other private or publicly funded clinics with different patient compositions. Additionally, given the high percentage of white men at the private clinic and the high percentage of black men at the public clinic, the group comparison results may also be due to differences in race/ethnicity as well as factors such as insurance status. Future validity testing should explore differences in race/ethnicity with populations of the same insurance and socio-economic status. Finally, we used a well validated anxiety measure as a starting point to develop anxiety items specific for prostate cancer screening and used cognitive testing to verify the initial content validity of the items. To develop unique items related to aspects about the healthcare decision, other qualitative methods like using focus groups to find out what people find anxious about healthcare decisions may provide a better insight for content domain and to generate a pool of items.

## Conclusions

Our six–item PCS-D anxiety measure could be used as a moderator in PCS decision aid studies. Our six–item PSA anxiety measure may also be used in PCS decision aid studies, although further invariance testing is needed to ensure accurate interpretation across populations that differ in race/ethnicity, level of education, and history of PSA testing, as well as insurance status. These two anxiety measures should be tested with other samples of men eligible for PCS. These anxiety measures are intended for research purposes and should not be used as a clinical diagnostic tool for anxiety. To develop a PCS anxiety measure with subscales related to the screening tests as well as the decision to undergo screening, researchers might use other qualitative techniques to generate an item pool of items. To minimize method variance, we recommend avoiding the use of similar wording across subscales.

Future research in decision aid studies should report the evidence for the validity of the factor structure and the invariance for affect measures. Because anxiety can influence effective decision–making and therefore modify the effects of decision aids, valid and reliable healthcare–specific anxiety measures are needed for other healthcare decisions other than PCS that involve uncertainty. Once such measures are created, the influence of anxiety on cognitive decision–making processes and differences across can be explored.

## Competing interests

The authors declare that they have no competing interests.

## Authors' contributions

SKL conceived the study, participated in the design of the study, performed the statistical analysis, and drafted the manuscript. PRS participated in the design of the study, helped perform the statistical analysis, and helped with the interpretation of the data. SWV participated in the design of the study and helped critically review the manuscript. ROM critically reviewed the manuscript and helped with the interpretation of the data. PDM participated in the conception of the study and helped draft the manuscript. RJV contributed to the conception of the measure, supplied the data, and helped draft and revise the manuscript. All authors read and approved the final manuscript.

## Pre-publication history

The pre-publication history for this paper can be accessed here:

http://www.biomedcentral.com/1472-6947/12/52/prepub
